# Herd Protection against Meningococcal Disease through Vaccination

**DOI:** 10.3390/microorganisms8111675

**Published:** 2020-10-28

**Authors:** Stephen A. Clark, Ray Borrow

**Affiliations:** Meningococcal Reference Unit, National Infection Service, Public Health England, Manchester M13 9WL, UK; ray.borrow@phe.gov.uk

**Keywords:** *Neisseria meningitidis*, meningococcal infection, meningococcal incidence, invasive meningococcal disease (IMD), IMD epidemiology, meningococcal vaccine, meningococcal vaccination

## Abstract

Reduction in the transmission of *Neisseria meningitidis* within a population results in fewer invasive disease cases. Vaccination with meningococcal vaccines composed of high weight capsular polysaccharide without carrier proteins has minimal effect against carriage or the acquisition of carriage. Conjugate vaccines, however, elicit an enhanced immune response which serves to reduce carriage acquisition and hinder onwards transmission. Since the 1990s, several meningococcal conjugate vaccines have been developed and, when used in age groups associated with higher carriage, they have been shown to provide indirect protection to unvaccinated cohorts. This herd protective effect is important in enhancing the efficiency and impact of vaccination. Studies are ongoing to assess the effect of protein-based group B vaccines on carriage; however, current data cast doubt on their ability to reduce transmission.

## 1. The Indirect Impact of Vaccination

The terms “Herd Immunity” or “Herd Protection” are used to describe a number of concepts relating to indirect protection of otherwise susceptible individuals against infectious disease due the immunity of other members of the population. There is a continued debate over the precise definitions of these terms; however, the concept of a population immunity threshold, beyond which transmission of an infectious agent is hindered and indirect protection is afforded to non-immune members, was described over a century ago [1,2,3,4]. It was not, however, until the late 1960s and 1970s and the introduction of the Intensified Eradication Programme against Smallpox that the concept was more widely discussed as a necessary part of achieving Smallpox eradication in the remaining affected parts of world. In 1971, Fox and colleagues argued that the prevailing ideas of single herd protective thresholds for large populations were too simplistic, as these populations were rarely homogenous in terms of susceptibility/immunity and likelihood for exposure/transmission [5]. Nevertheless, the authors argued that the concept could be utilised through targeted vaccination of subgroups identified as more likely to transmit the agent, thus maximising the indirect benefits of vaccination. Herd protection through vaccination is most clearly observed against agents that are transmitted from person-to-person with no extra-human sources of infection. Consequently, the diseases caused by bacterial pathogens indigenous to the human respiratory tract are particularly vulnerable to any reduction in person-to-person transmission. Along with *Haemophilus influenzae* type b (Hib) and *Streptococcus pneumoniae*, *Neisseria meningitidis* (the meningococcus) is one such obligate human pathogen.

## 2. The Meningococcus: A Human Pathogen

Colonisation of the pharyngeal tissues is considered to be the natural state for the meningococcus, despite its propensity to invade the host epithelium and cause infections ranging from transient, non-systemic bacteraemia to devastating systemic shock and meningitis [6]. The intrinsic environmental, host and bacterial factors that trigger the transition from a stable carriage state to infection are not yet fully understood; however, a number of virulence determinants have been shown to facilitate binding of the host epithelium, the formation of microcolonies and subsequent paracellular or transcellular invasion into the bloodstream and, in some cases, across the blood–brain barrier [7,8]. The ability to express capsular polysaccharide is perhaps one of the most important virulence factors for invasion-prone strains. The differing chemical composition of the polysaccharides expressed allows for the division of strains into 12 capsular groups, six of which (A, B, C, W, X and Y) cause the vast majority of invasive cases globally [9]. The importance of these innate bacterial factors is demonstrated by the fact that the vast majority of IMD cases amongst immune-competent individuals are caused by strains belonging to a limited number of hyper-virulent lineages [10].

Immune protection against IMD can be acquired naturally through the production of serum bactericidal antibodies in response to the carriage of *N. meningitidis* (particularly non-pathogenic strains) or by transfer to maternal antibodies to infants. IMD most commonly occurs among those <2 years, an age group in which carriage rates are very low. This lack of contact with *N. meningitidis* in early life delays the development of natural immunity and leaves individuals susceptible to disease once any maternal antibodies have waned (typically at around 6 months of age) [11]. Such immunity gaps within the population and the disease that consequently follows has triggered the development of several effective meningococcal vaccines. Here we review the impact of these vaccines on the carriage and transmission of *N. meningitidis* and demonstrate the importance of herd protection in the reduction in the IMD burden around the world.

## 3. Meningococcal Carriage in the Population

Transmission of *Neisseria meningitidis* occurs through air droplets from respiratory secretions. Unlike other bacterial pathogens that inhabit the pharyngeal tissues (e.g., the pneumococcus and Hib), meningococcal carriage is relatively low among infants and young children. Within developed countries at least, carriage rates tend to increase throughout childhood and peak among adolescents and young adults [12]. Age is not the only determinant of transmission potential. Social behaviours, such as kissing, smoking, crowding (e.g., in bars and nightclubs) and living in close proximity have all been implicated in increasing person-to-person spread [13]. For these reasons, high rates of disease are typically observed within semi-closed institutions, such as higher education establishments, care homes and military installations. In 2017, Mandal et al. found that English University students had an 11-times higher risk of invasive meningococcal disease than non-students in the same age group [14]. Institutions are not the only setting in which risk of transmission is greater. The crowded conditions typically experienced during Hajj and Umrah, as well as other mass gatherings, can also facilitate transmission and lead to outbreaks [15,16].

## 4. The Impact of Plain Polysaccharide Vaccination on Carriage

It was a high disease burden in US military bases in the 1960s that galvanised the push to develop the first immunogenic meningococcal vaccines [17,18]. Outbreaks of group A and C disease led to the isolation of high molecular-weight capsular polysaccharide antigens that provided limited group-specific protection in older children and adults, although infants and young children did not generate sufficient antibody responses for reliable protection [19]. Moreover, the impact of these “plain” polysaccharide vaccines on nasopharyngeal carriage of meningococci is likely to be minimal. There is a small number of studies, many of which were performed in military recruits, that have reported statistically significant reductions in carriage following polysaccharide vaccination; however, many of these studies suffer from methodological issues [20]. Almost all reliable studies reported no change or an increase in carriage following vaccination, in particular those in civilian populations [20,21,22]. The failure of these vaccines to impact carriage is likely to be due to the limited immune response. The meningococcal capsular polysaccharide is a T-cell independent antigen, meaning that it that does not bind MHC-II and elicit antigen presentation or T-cell activation. The immune response to these antigens is therefore typically restricted to IgM antibody isotypes and low memory B cell generation [23].

## 5. The Development and Impact of Group C Polysaccharide Conjugate Vaccines

The 1980s saw the development of conjugate vaccines against Hib disease. These vaccines utilise high molecular weight polysaccharide polyribosyl ribitol phosphate (PRP) chemically conjugated to a carrier protein such as tetanus toxoid, diphtheria toxoid or diphtheria CRM-197 mutant [24]. The inclusion of a carrier protein yields a T-cell dependent immune response characterised by B-cell-T-cell interaction, proliferation of CD4+ T helper cells and associated cytokine production. This augmented immune response results in higher avidity antibody production, isotype switching and induction of immunological memory [25,26]. The introduction of Hib vaccine programmes in the 1990s led to drastic reductions in Hib disease incidence in both vaccinated and non-vaccinated cohorts, indicating a herd protective vaccine effect [27]. The reduction of Hib carriage has been suggested to be due to the transudation of PRP-specific IgG into the pharyngeal tissues among vaccinees with high IgG titres [27,28,29].

Following the success of Hib conjugated vaccines in the early 1990s, vaccine developers began to question whether the same approach could be used to improve meningococcal polysaccharide vaccines, which suffered similar disadvantages to their unconjugated Hib counterparts. This effort was bolstered by the increase in group C disease due to clonal complex (CC) 11 across Canada and Europe in the mid-1990s [30]. In the UK, a group C meningococcal conjugate (MCC) vaccine development programme involving a collaboration between public health laboratories, vaccine manufacturers and academia resulted in the development and licensure of three MCC vaccines—two containing the CRM-197 carrier protein and the other using tetanus toxoid (TT) [30]. The UK MCC vaccine programme was launched in November 1999 with vaccination of infants at 2, 3 and 4 months, followed by a catch-up campaign for those up to the age of 18 years. In 2002, this was extended to include those up to 25 years. The rationale behind the catch-up campaign was to provide direct protection to an adolescent age group that had relatively high incidence of group C disease, whilst also attempting to reduce carriage among those with the highest rates of carriage [30].

**Figure 1:**
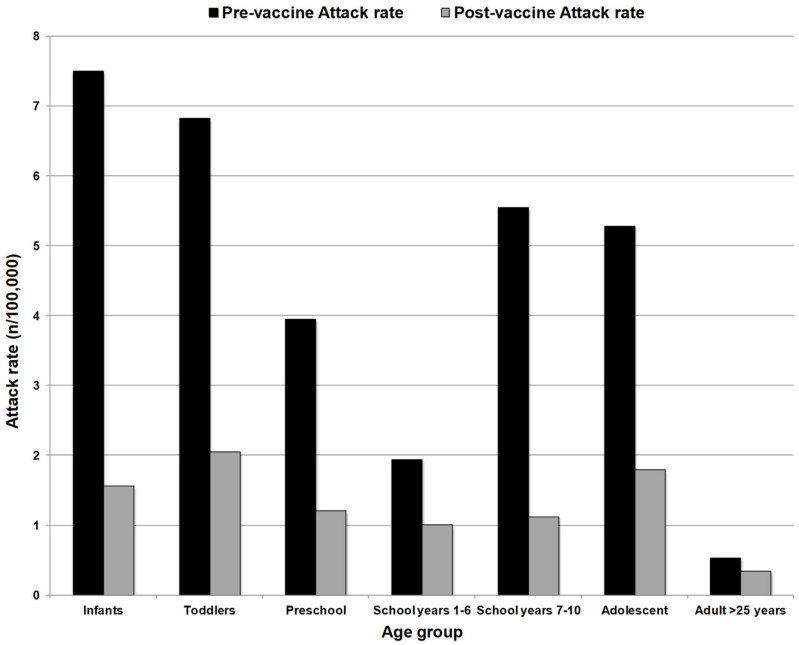
Group C attack rate (cases per 100,000 population) among unvaccinated people before MCC introduction (1998/99) and after vaccine introduction (2001/2002). Substantial reductions were observed across all age groups despite lack of direct protection, indicative of a herd protective effect. Adapted from Ramsay et al. (2003).

The overall incidence of group C disease in England and Wales reduced from 1.85/100,000 in 1998/1999 to 0.12 in 2003/2004 and vaccine effectiveness was found to be between 83–100%. Reductions in group C disease were also observed in those aged >25 years who had not been vaccinated, with incidence reducing from 0.55/100,000 to 0.11/100,000 between 1998/1999 and 2003/2004 [31,32]. Further evidence of herd protection was provided in an analysis by Ramsay et al. in 2003. They demonstrated a substantial reduction in the MenC attack rate among unvaccinated cohorts among all age groups, following mass vaccination with MCC in the UK (Figure 1, [33]).

Similarly, following the introduction of MCC vaccination in Spain in 2000, a reduction in incidence risk ratio was observed across all age groups regardless of vaccinations status [34]. In the Netherlands, up to a 50% reduction in group C disease incidence was observed among those above the age of vaccination following the introduction of adolescent vaccination catch-up programme [35]. Canada also saw a similar impact following MCC introduction [36].

In the UK, sustained reductions in disease rates were seen despite demonstrable waning of antibodies in infants within 12 months after vaccination [37]. These findings further demonstrate the importance of herd protection in suppressing disease as direct protection in these age groups is short lived. As predicted, this herd protective effect was a result of a reduction in strain-specific carriage among the vaccinated. Carriage of the causative group C-expressing CC11 strain among 15–17 year olds reduced by 94% between 1999 and 2001 [38].

In France, MCC vaccination was introduced in 2010, targeting 12-month-olds, as well as a catch up programme for those <25 years old [39]. The strategy was to directly immunise toddlers whilst relying on herd protection from the adolescent campaign to protect those <1 year old and older age groups. Interestingly, following vaccine introduction, increases in group C disease were reported among those <1 year old and other unvaccinated age groups. It was concluded that low vaccine uptake (<40%) was the reason for a lack of indirect protection, highlighting the importance of high coverage in order to disrupt person-to-person transmission [39].

## 6. The Use of Quadrivalent Polysaccharide Conjugate Vaccines

In the mid-2000s, the development of ACWY quadrivalent conjugate vaccines was driven by the prevalence of these four serogroups among invasive strains globally. In recent years, group W strains belonging to CC11 have caused several related outbreaks across the world. In the early 2000s, cross-border transmission of a group W CC11 strain among returning Hajj pilgrims caused outbreaks in Europe, North Africa and Asia [40]. Since 2009, the global spread of a distinct but related group W CC11 strain (W:CC-11) has resulted in outbreaks in Europe, Australia and the Americas [41,42]. Increases in group Y disease have been reported in Europe in recent years, whilst group A disease had been, until recently, the predominant cause of IMD in sub-Saharan Africa [43,44].

There are currently three ACWY conjugate vaccines licenced for use in different countries/regions and age groups. Two of these vaccines feature diphtheria toxoid (D) or the CRM-197 mutant as the carrier protein, and the remaining vaccine is conjugated with TT [45]. Since the mid- to late 2000s, ACWY vaccines have been recommended for adolescents in Canada and the United States. Greece and Austria also recommended adolescent doses in 2011 and 2012, respectively, and Argentina has recently added an ACWY infant and adolescent programme to its national schedule [46,47]. Chile included the ACWY vaccine to its national programme in 2012 for infants and young children [48]

In the UK, ACWY conjugates (ACWY-TT and ACWY-CRM-197) were added to the national immunisation schedule in 2015 primarily in response to the increase in hyper-invasive group W CC11 disease. School children aged 13–14 were targeted and a limited catch-up programme for those up to 18 years (plus students <25 years) was also introduced [49]. The rationale was to provide direct protection against group W disease to those approaching the age of high risk (especially university students), whilst also generating indirect herd protection to younger age groups against both groups W and C disease. This vaccine replaced an adolescent MCC dose in this age group, which was particularly crucial due to the gradual reduction and ultimate removal of MCC doses in the infant schedule.

**Figure 2:**
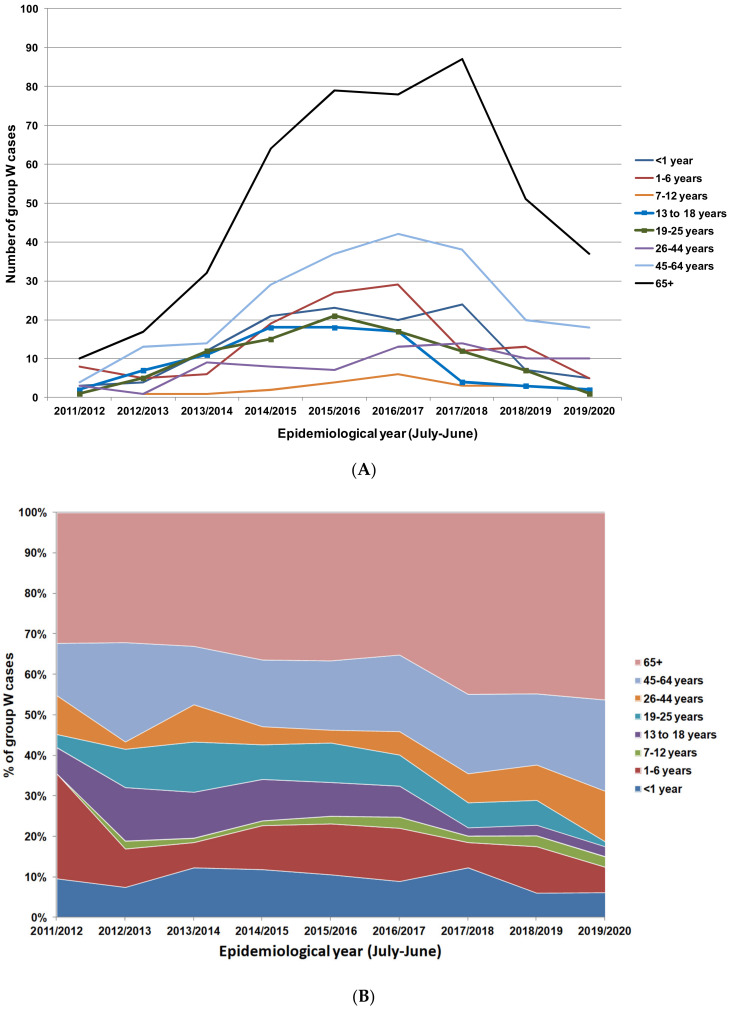
(**A**) Group W IMD cases in England from 2011/2012 to 2019/2020 stratified by age groups. Age groups offered vaccines are highlighted with square markers (Public Health England: unpublished data). (**B**) Percentage distribution of group W IMD cases in England from 2011/2012 to 2019/2020 by age group.

Initial data showed a modest reduction in group W cases among vaccinees; however, this was against a background of low vaccine uptake (36.6%) in the first year of the campaign [49]. This low uptake was primarily due to the lack of a school-based programme in that first year. Uptake increased markedly in subsequent years and had reached 85–90% by 2018/2019 [50]. This resulted in a delay in any substantial reduction in cases among vaccinated age groups until 2017/2018 (Figure 2A Public Health England: unpublished data). The most substantial decreases among the unvaccinated cohorts (65+ years in particular) were observed from 2018/2019 onwards, probably indicative of a further delay before transmission reduction translated into a reduction in disease in unvaccinated groups. Between 2017/2018 and 2019/2020, cases in the 65+ years age group reduced by over 50% (Figure 2A). The proportion of group W cases attributed to the vaccinated age groups (13–25 years) had reduced from 18.2% in 2014/2015 to 3.4% in 2019/2020, whilst the share of cases among non-vaccinated age groups had increased despite an overall reduction in cases in these cohorts (Figure 2B, Public Health England: unpublished data). This pattern confirms that direct vaccination results in a more immediate and sizable reduction in cases in relation to indirect herd protection. In 2020, Ladhani et al. used Poisson modelling to estimate the impact of herd protection against group W cases following the introduction of the ACWY in adolescents. They estimated that the vaccine introduction prevented between 114 and 899 group W cases in those aged under 5 years [51].

Conversely, an ACWY vaccination campaign targeting young children in Chile failed to yield any detectable reduction among unvaccinated age cohorts, despite a 92.3% reduction in group W cases in the vaccinated children [48]. These findings concisely demonstrate the importance of targeting age groups with high levels of carriage in order to confer indirect protection across the population.

## 7. The Impact of MenAfriVac Group A Conjugate Vaccine on Disease and Carriage in Sub-Saharan Africa

The sub-Saharan region of Africa suffered devastating group A IMD outbreaks for decades. These epidemics typically coincided with the Winter season in which the dry, dusty conditions can lead to a weakening of the pharyngeal epithelium and increase the likelihood of invasion by hyper-virulent meningococcal strains. For much of this time, the lack of an affordable group A conjugate vaccine led to a reliance on plain polysaccharide vaccines. The inability of these vaccines to provide long-lasting immunity or indirect protection resulted in the adoption of reactive vaccination strategies to outbreaks as they occurred. These reactive campaigns were difficult to initiate quickly enough to prevent disease in the early stages of epidemics and they didn’t prevent recurrence of epidemics in subsequent years [52].

In order to move to a preventative vaccination strategy and to eliminate group A disease from the region, the Meningococcal Vaccine Project (MVP) was formed in 2001 with the aim of developing an affordable group A conjugate vaccine for sub-Saharan Africa. The MVP, a collaboration between the World Health Organization, Program for Appropriate Technology in Health (PATH) and the Serum Institute of India, achieved this aim in 2010 with the licensure of PSA-TT (MenAfriVac) [53]. MenAfrivac (PSA-TT) is conjugated to TT and vaccination of the first communities began in 2012 with those aged between 1–29 years eligible for vaccination. By 2018, over 300 million people across 22 countries had been immunised with high coverage in most countries [54]. The overall impact on group A disease has been dramatic. In 2017, Trotter et al. analysed surveillance data across nine sub-Saharan countries and reported a 99% reduction in group A disease incidence in fully vaccinated populations [55].

As a conjugate vaccine, the possibility for indirect protection through disruption in carriage was a strong driver in the development of PSA-TT. Due to the high disease burden in the sub-Saharan countries, meningococcal carriage surveys have been performed in this area of the world for many years [21,56]. Many of these studies were performed during outbreaks and so the reported meningococcal carriage rates vary widely and in some cases can be as high as 25–30% [21]. The age distribution can be equally as variable; however, a general trend suggests that the carriage prevalence in these countries is likely to peak slightly earlier than in industrialised countries, with higher rates observed in those around 5–14 years old [21,57].

The impact of PSA-TT on carriage was assessed through a series of carriage surveys across the continent before and after mass vaccination. Pre-vaccination surveys across five countries observed an overall meningococcal carriage rate of 3.4%, although wide variation was observed across the different sites and population demographics [58]. Group A carriage was relatively low with a number of surveys failing to isolate/identify any group A strains [56]. In Burkina Faso, group A carriage was reported to be 0.24–0.62%, whilst in the Mandelia district of Chad, which was experiencing a group A outbreak at the time of sampling, only 0.6–0.7% of participants carried a group A strain [59,60].

In addition to a dramatic reduction in disease rates, PSA-TT has also been shown to have an equally substantial impact on group A meningococcal carriage. In the survey in Mandelia (Chad) six months following mass vaccination with PSA-TT, the group A carriage rate had reduced substantially to 0.02%, with only one group A isolate recovered among almost 5000 people [60]. Interestingly, in the survey performed 2–4 months prior to mass vaccination, seven group A strains were isolated from those among those aged <1 or >30 years (age groups not eligible for vaccination). In comparison, no group A strains were isolated in these non-eligible age groups in the post-vaccination survey. Although the numbers are small, these data suggest reduced transmission from vaccinated to non-vaccinated populations, indicative of the herd protection effect. A very similar impact was observed in Burkina Faso with only one group A isolate isolated from almost 5000 people (0.02%) sampled two years after mass vaccination with PSA-TT [61].

Whilst the reduction in carriage following vaccination is the key driver of herd protection, the wide target age range for vaccination (1–29 years) and high vaccine uptake in the MenAfriVac campaign means the precise contribution of herd protection to the reduction of group A disease is difficult to ascertain. Nonetheless, very few group A cases are now observed in vaccinated regions in all age groups, indicating herd protection of infants and older adults who were not eligible for vaccination and of any vaccine recipients who failed to respond to the vaccine immunologically.

## 8. Potential for Herd Protection Using MenB Vaccines

Due to the lack of immunogenicity exhibited by the group B polysaccharide antigen, the first effective vaccines against group B disease were composed of outer membrane vesicles (OMVs) derived from specific outbreak strains [62]. OMV vaccines have been successful at controlling outbreaks in Cuba, Europe and New Zealand. The immunodominance of PorA within these formulations (the VR2 epitope in particular) results in a narrow, strain-specific coverage and, despite efforts to broaden this coverage through the inclusion of additional antigens, OMVs have not yet been utilised to protect against diverse endemic strains [62,63].

Partly due to the sporadic use of these vaccines, few large-scale studies have been performed to assess the impact on carriage and the findings are often conflicting. Early studies in children and adolescents in Norway, Chile and Iceland found no detectable reduction of meningococcal carriage following vaccination with different OMV formulations [64,65,66]. In 2008, Holmes et al. reported a reduction in carriage from 40.4% to 21.1% among 57 adolescent students following vaccination with MeNZB (B:4:P1.7-2,4:cc41/44), whilst no significant change was observed in the unvaccinated group. In this case, carriage of the target/outbreak strain was only detected among the unvaccinated cohort [67]. In 2013, Delbos et al. assessed meningococcal carriage among children following a cc32 outbreak in Normandy. They reported significantly lower carriage among children who had received at least one dose of the MenBVac OMV vaccine (B:15:P1.7,16:cc32) compared to unvaccinated children (0.31% vs. 2.1%), although baseline carriage was not assessed [68]. Interestingly, all but two of the 16 carriage strains isolated amongst the unvaccinated cohort (88%) were non-cc32 strains, whilst only one meningococcal isolate (non-cc32) was isolated from the vaccinated group [68]. These findings suggest that the vaccine elicited carriage clearance and/or prevented the acquisition of a wide array of diverse meningococcal strains. This contrasts markedly with the strain-specific SBA responses typically produced by MenBVac and other OMV vaccines. Although, as part of the same study, Delbos and colleagues also produced data from a respiratory mouse model, suggesting that OMVs may induce a broader and more cross-reactive IgG repertoire within the respiratory tract than observed in the serum [68].

The most recent generation of licenced group B vaccines features recombinant protein antigens with cross-reactive attributes that allow for a broader strain coverage in order to reduce endemic group B disease. rLP2086 (Trumenba^®^, aka MenB-FHbp, Pfizer Inc, Philadelphia, PA, USA) was licenced for use in the US in 2014 and in Europe and Canada in 2017, and consists of two distinct lipidated variants of Factor H-Binding Protein (fHbp) [69]. 4CMenB (Bexsero^®^, GlaxoSmithKline Vaccines, Siena, Italy) was licenced in Europe, Canada and Australia in 2013 and the US in 2015, and contains the MeNZB OMV preparation, along with four recombinant proteins (including fHbp, Neisserial Heparin Binding Antigen (NHBA) and *Neisseria* Adhesin A (NadA)), presented as two fusion proteins [70,71]. Both have since been used for at-risk groups and in response to a number of group B outbreaks [72,73,74,75].

Since 2015, 4CMenB has been included in the UK national infant immunisation programme with three doses administered to infants at 2, 4 and 12 months. Whilst the introduction of 4CMenB has been attributed to a substantial reduction in group B disease in the UK, its use in infants is not expected to yield any herd protective effects as carriage within this age group is low [12,76]. In many countries, inclusion of the vaccine into infant vaccination programmes has not been deemed cost effective due to the high cost of the vaccine and the relatively low incidence of group B disease. However, modelling has shown that, if carriage/transmission was interrupted following vaccination of adolescents with 4CMenB, the cost effectiveness of the vaccine could be substantially improved due to an increase in the number of cases prevented through herd protection [77,78,79,80,81]. In 2018, South Australia included 4CMenB in its infant routine programme (2 + 1 schedule) and, in 2019, also introduced it into the adolescent school-based programme (2 doses, 14–15-year-olds). Enhanced epidemiological monitoring is ongoing to detect any evidence of herd protection.

The first attempt to determine the potential impact of 4CMenB on carriage was made by Read and colleagues in 2014. In a phase 3 randomised controlled trial, almost 3000 English university students (18–24 years) were vaccinated with two doses of either 4CMenB or a control vaccine (Japanese encephalitis), or a single dose of MenACWY-CRM followed by a placebo [82]. Pharyngeal carriage was assessed at pre-vaccination and every two months up to a year post vaccination. At one month, following the second dose, no significant differences in carriage prevalence were seen between the three groups (4CMenB, MenACWY-CRM and control). After combining the data for all time points from four to twelve months, a small but statistically significant reduction was observed in the carriage of all meningococci in the 4CMenB group; however, the difference was not significant for group B strains only [82]. The authors also reported no difference in the acquisition of meningococci compared to the controls at the same aggregate time points; however it was noted that much of the acquisition (particularly group B) occurred at earlier time points and so vaccination may have been too late to demonstrate a more defined effect. Similar results were observed for the MenACWY-CRM group after one dose, with a significantly lower acquisition of group Y strains in particular [82].

A series of group B outbreaks in the US in the mid-2010s provided the opportunity to assess the impact of these vaccines on carriage in a real-life setting. Soeters and colleagues performed a carriage assessment following an outbreak at a university in 2015. Participants were offered three doses of rLP2086 with carriage assessed periodically at each vaccination visit and then 6 months following the third dose. No reduction in carriage of any meningococci was observed among the 27% (169/626) of participants who received the full three-dose vaccine course, or indeed fewer doses [83]. In 2017, McNamara et al. assessed carriage impact following the use of 4CMenB or rLP2086 in university students following a group B university outbreak in North West US. The findings showed no association between either vaccination and carriage reduction; however, at the final assessment six months after third dose, of the participants reporting vaccination status, only 3.6% of had received the full course of 4CMenB (two doses) and only 6.2% of these participants had received the full three-dose course of rLP2086 [84].

In terms of assessing the impact of 4CMenB on carriage, perhaps the most conclusive data produced thus far are those of the 2017 “B Part of It” trial in South Australia. The carriage study recruited approximately 25,000 adolescents, with one group receiving two doses of 4CMenB two months apart and an unvaccinated control group [85]. Pharyngeal swabbing took place at baseline and at the 12-month time point. The results indicated no significant differences between the vaccinated and unvaccinated groups in terms of carriage rates at 12 months or acquisition of carriage over the study. Moreover, the carriage rate of any meningococci increased two-fold from baseline to 12 months. These findings provide strong evidence that 4CMenB is ineffective at clearing carriage or preventing meningococcal transmission [85]. Given that 4CMenB also contains an OMV preparation, these results also cast doubt on the ability of OMV vaccines generally to impact carriage.

At the time of writing, a large national meningococcal carriage study is ongoing in the UK to assess the impact of both 4CMenB and rLP2086 on carriage in sixth form adolescents. The “Be on the Team” study has recruited approximately 24,000 students to receive one of the two vaccines, or a control vaccine, with carriage being assessed at baseline and 12 months [86].

## 9. Mechanisms of Protection against Meningococcal Carriage

As with Hib and Pneumococcal conjugates, the precise mechanism by which meningococcal conjugate vaccination leads to protection against carriage has yet to be fully understood. It may be the case that multiple mechanisms play contributory roles in this protective response; however, mucosal antibodies, specifically IgA and IgG, are likely to be key drivers through interactions with colonising strains.

IgG is perhaps the most versatile of the antibody classes and isotype switching from IgM to IgG is one of key advantages of the T-cell dependent antigen response observed following conjugate/protein-based vaccination. IgG are particularly efficient at inducing bactericidal activity through the activation of the classical complement pathway, but also promote opsonophagocytic activity and PMN-mediated respiratory burst [87]. IgA typically plays a prominent role in the mucosal tissues and, whilst it cannot bind C1q and cause complement-mediated lysis, it has been implicated in monocyte-mediated lysis and PMN-mediated respiratory burst against meningococci in vitro [87,88].

Few studies have directly compared differences in the mucosal immune responses between vaccination with plain polysaccharide and conjugate vaccines. Two studies by Zhang and colleagues investigated the salivary antibody response to plain and conjugated group A + C vaccines and found that increases in polysaccharide-specific salivary IgA were more prominent following vaccination with plain polysaccharides compared to conjugate vaccines [89,90].

Several studies have measured both salivary and serum antibodies and, overall, the correlations between the two sites appear to be stronger for IgG than IgA, suggesting that much of the mucosal IgG is derived from the serum via transudation [89,91,92,93,94]. Similarly, Borrow et al., (1999) found a strong correlation between salivary polysaccharide-specific IgA and secretory component IgA, indicating that the mucosal IgA response to meningococcal vaccination is largely a result of antibody production within the local tissues [91].

Interestingly, the presence of IgA has been shown to inhibit the complement-mediated bactericidal activity of IgG in vitro [87,95,96]. This inhibition can be overcome as the relative IgG concentration increases [87]. If this inhibitory activity occurs in the pharyngeal tissues, the signature higher serum IgG and lower mucosal IgA typically observed following conjugate vaccination (vs. plain polysaccharides) may play an important role in clearance by reducing the inhibition of IgG-mediated complement activity in the nasopharynx [89,97].

In 2017, Vianzon et al. studied the effect of mucosal IgG derived from immunisation with different meningococcal vaccines on adherence to and invasion of human epithelial cells in vitro. They found that IgG derived from MCC vaccination reduced the invasion of epithelial cells by preventing the shedding of the capsule (previously observed among invading strains) through capsular blebbing [98]. Their data also suggested that IgG may also reduce microcolony dispersal, making carried strains more susceptible to clearance and less likely to spread via aerosol droplets, thus reducing transmission. Crucially, these effects were only observed for IgG derived from MCC vaccination and not those from vaccination with plain polysaccharides, group B OMVs or fHbp-based vaccines [98]. These preliminary data suggest that the specific epitope to which mucosal antibodies bind may be a greater determinant of carriage-reducing capability than simply the amount of the antibody within the nasopharynx.

## 10. Conclusions

As an intrinsic human pathogen, *N. meningitidis* is particularly susceptible to any disruption in person-to-person transmission. Whilst the stable carriage state can last for several months, a breakdown in these transmission chains will inevitably result in a reduction in the spread of hypervirulent strains and cases of disease within the population.

The ability of meningococcal conjugate vaccines to disrupt transmission through a population has been demonstrated in several contexts since the development of the first MenC conjugates in the 1990s. If the cohorts with the highest carriage rates are targeted with vaccination, typically adolescents, the consequent prevention of onwards transmission leads to a substantial reduction in disease incidence amongst the unvaccinated within the population. This indirect effect allows for more strategic and efficient use of vaccines by targeting these high carriage age groups only; however, the vaccine coverage/uptake in these age groups must be high in order to successfully hinder transmission. The precise mechanisms by which conjugate vaccines effect this disruption are still elusive; however, the superior IgG response is likely to play a key role.

Mounting data on the current protein-based group B vaccines indicate that they are unlikely to reliably reduce carriage or prevent acquisition. The lack of knowledge concerning the exact mechanisms by which carriage is reduced is likely to hinder the development of group B vaccines that can provide herd protection.
